# Taohong siwu decoction attenuates AIM2 and NLRC4 inflammasomes by ameliorates deoxyribonucleic acid damage after ischemic stroke

**DOI:** 10.3389/fphar.2022.954867

**Published:** 2022-08-12

**Authors:** Ni Wang, Furui Chu, Lijuan Zhang, Changyi Fei, Chao Yu, Sujun Xue, Yongzhong Wang, Ling Fang, Daiyin Peng, Xianchun Duan, Weidong Chen

**Affiliations:** ^1^ Department of Pharmacy, The First Affiliated Hospital of Anhui University of Traditional Chinese Medicine, Hefei, China; ^2^ School of Pharmacy, Anhui University of Chinese Medicine, Hefei, China; ^3^ MOE-Anhui Joint Collaborative Innovation Center for Quality Improvement of Anhui Genuine Chinese Medicinal Materials, Hefei, China; ^4^ Anhui Province Key Laboratory of Chinese Medicinal Formula, Hefei, China; ^5^ Key Laboratory of Xin’an Medicine (Anhui University of Chinese Medicine), Ministry of Education, Hefei, China; ^6^ Department of Pharmacy, The First Affiliated Hospital of Anhui Medical University, Hefei, China

**Keywords:** Taohong Siwu Decoction, ischemic stroke, AIM2, NLRC4, inflammasomes

## Abstract

Taohong siwu decoction (THSWD) has been shown to have a therapeutic effect on ischemic strokes (IS). However, it is not clear to us whether THSWD reduces deoxyribonucleic acid (DNA) damage after stroke and reduces the inflammatory response caused by the damage. Therefore, we constructed an IS model (I/R) in rats and performed oxygen-glucose deprivation/reoxygenation (OGD/R) on BV2 cells. Then ELISA, immunofluorescence staining, immunohistochemistry staining, and RT-qPCR were performed to detect the expressions of absent in melanoma 2 (AIM2), NLRC4, and Caspase-1 inflammasomes and other inflammatory factors. Experimental stroke causes DNA damage, and we found that the aforementioned inflammasomes as well as inflammatory factors were significantly inhibited after treatment with THSWD by comparing the model group with the model administration group. In addition, we examined the expression of AIM2, NLRC4, and Caspase-1 in BV2 cells of OGD/R and found that the expression of the aforementioned inflammasomes was significantly decreased in OGD/R by administration of THSWD-containing serum. Our data suggest that THSWD can reduced DNA damage after stroke as well as the inflammatory response caused by the damage.

## Introduction

Ischemic stroke (IS) is caused by the occlusion of the arteries in the brain, causing brain damage and inflammatory response (Pluta et al., 2021). Brain damage caused by IS includes excitatory toxicity, oxidative stress, neuroinflammation, apoptosis, and other phenomena (Sekeljic et al., 2012; Radenovic et al., 2020). Damaged brain areas get progressively worse as blood flow decreases (Majid, 2014). The main principles of treatment for IS include dissolving blood clots and restoring blood supply, but blood recirculation can cause cerebral ischemia-reperfusion injury (Omoto et al., 2022). Among them, deoxyribonucleic acid (DNA) damage and inflammatory response are considered to be important pathological mechanisms of cerebral ischemia-reperfusion injury (Dasdelen et al., 2021; Franke et al., 2021).

Oxidation-induced secondary damage occurs during IS, resulting in oxidative damage to DNA (Lorente et al., 2021). Too much production and release of pro-inflammatory cytokines in response to DNA damage severely affect the ability of cells to regenerate. Among them, NLRP3 and AIM2 inflammasomes are the main inflammasomes involved in DNA damage and associated cytokine release (Cinat et al., 2021). Inflammasomes have now been shown to be key mediators in triggering inflammation after IS, and it is a novel multimeric protein complex (Mohamed et al., 2015). NLRC4 is a member of the NLR family, which activates caspases, forms inflammasomes, and participates in the regulation of inflammatory responses (Sundaram and Kanneganti, 2021). Absent in melanoma 2 (AIM2) is derived from damaged cells and can trigger an inflammatory response by recruiting caspase-1 (Zhang et al., 2020). It has been shown that AIM2 inflammasome and NLRC4 inflammasome expression is upregulated after IS. AIM2 inflammasome leads to brain damage and cognitive impairment after chronic stroke in mice (Habib et al., 2020).

Taohong Siwu Decoction (THSWD) was first recorded in “Yizong Jinjian” written by Wu Qian. It is a classic formula for activating blood circulation and resolving blood stasis, with the effect of nourishing blood circulation, eliminating blood stasis, and creating new blood (Xia et al., 2021). Zhang et al. (2018) explained the mechanism of THSWD intervention in acute blood stasis model rats based on liquid chromatography with quadrupole time-of-flight mass spectrometry (LC/Q-TOF-MS) of urine metabolomics, and they found that the intervention mechanism may be mainly related to the regulation of lipid metabolism and amino acid metabolism. It has been shown that THSWD reduces the activation level of NLRP3 inflammatory vesicles in MCAO/R rats, downregulates GSDMD, and inhibits cellular scorching (Wang et al., 2020). Using network pharmacology, Pan et al. (2022) conducted a preliminary exploration of the mechanism of action of THSWD for IS and found that THSWD reversed the high expression levels of C1qb, C1qc, C3ar1, C5ar1, and Cfd proteins and inhibited the inflammatory response after cerebral ischemia. Our group’s previous study showed that THSWD could reverse the expression of inflammation in IS and reduced the pathological manifestations (Wang et al., 2021). However, it is not clear to us whether THSWD reduces DNA damage after stroke and reduces theinflammatory response caused by the damage. Experimental stroke causes DNA damage. We observed DNA damage by gavage of THSWD in stroke rats and assessed the effect of THSWD on AIM2 and NLRC4 inflammasomes vesicles in rat brains after stroke. In addition, to provide ideas for further studies of THSWD, we evaluated the expression and regulation of AIM2 and NLRC4 inflammasomes in BV2 cells after oxygen-glucose deprivation/reoxygenation by THSWD-containing serum.

## Materials and methods

### Herbal medicine and animals

The specific information on herbs is shown in Table 1. According to the proportion of herbs in Table 1, the medicinal materials were first extracted with 10 times the amount of water for 2 h and filtered, and the filtrate was stored. The filtrate was extracted with eight times the amount of water for 1.5 h, filtered, and then combined twice and concentrated (Wang et al., 2021). Fifty male Sprague–Dawley (SD) rats were obtained from the Experimental Animal Center of Anhui University of Traditional Chinese Medicine. The rats were confined in a room with appropriate temperature and humidity and given feed and water on a daily basis. All animal experiments were approved by the Animal Experiment Ethics Committee of Anhui University of Chinese Medicine (license number: LLSC20160336).

**TABLE 1:** Constituents of THSWD.

Components	Part used	Proportion	Batch number
Bai Shao (BS) (*Paeonia lactiflora* Pall.)	Dried root	3	17050301
Shu Dihuang (SDH) [*Rehmannia glutinosa* (Gaertn.) DC.]	Dried root	4	17042501
Dang Gui (DG) (*Angelica sinensis* (Oliv.) Diels)	Dried root	3	16070501
Chuan Xiong (CX) (*Conioselinum anthriscoides* ‘Chuanxiong’)	Dried rhizome	2	17061601
Tao Ren(TR) [*Prunus persica* (L.) Batsch.]	Dried ripe seed	3	17033101
Hong Hua (HH) (*Carthamus tinctorius* L.)	Dried flower	2	17041401

### Animal model establishment and grouping

After 1 week of rats rearing, based on the literature by Longa et al. (1989), a rat middle cerebral artery occlusion model (I/R) was constructed in SD rats. The specific operations are as follows: we made a hole with scissors 1 cm from the bifurcation of the common carotid artery in the rat; inserted a fish line into the hole, approximately 18–22 mm; and partially withdrew the line after 2 h to restore blood flow and then sutured the wound. The animals were scored for neurological function within 12 h after the establishment of the animal model, and the rats were randomly divided into the model group and the drug administration group. A Zea Longa 5-point scale was used for neurological function scores. The specific scored criteria are shown in Table 2. Longa scores of 1–3 were considered appropriate for inclusion in the study.

**TABLE 2:** Neurological function score.

Score	Symptoms seen in rats
0	Moved normally
1	The left front paw could not be successfully extended
2	Turned to the left when crawling
3	The rat’s body was skewed to the left when crawling
4	Unconscious, unable to crawl

Rats were assigned into three groups with 10 rats each: Sham, I/R, and I/R + THSWD. In the I/R + THSWD group, 9 g/(kg-d) of THSWD was continuously administered intragastrically for 7 days, and in the remaining groups, the same amount of normal saline was administered intragastrically.

### Preparation of hippocampal cell suspension

The hippocampus was removed from the rat brain and put into a Petri dish containing PBS solution, and then, the hippocampus was transferred to a 1.5 ml PE tube and digested with 0.25% trypsin 1 ml in a 37°C water bath for 10 min. After the digestion was finished, serum was added, filtered through 300 mesh nylon mesh, and centrifuged at 1000 rpm for 5 min, and the precipitate was cells.

### Taohong siwu decoction drug-contained serum intervention

SD rats were assigned into two groups with 10 rats each: the normal group and the drug-containing serum group. In the drug-contained serum group, 9 g/(kg-d) of THSWD was continuously administered intragastrically twice a day for 7 days, and in the normal group, the same amount of normal saline was administered intragastrically. Anesthetized rats had their blood taken from the abdominal aorta, centrifuged, and passed through the filter membrane.

### Oxygen-glucose deprivation/reoxygenation model

BV2 cells were maintained in a humidified environment at 37°C with 5% CO_2_. The cells were cultured in Dulbecco’s modified Eagle medium (DMEM, Gibco Biotech, United States) supplemented with 10% fetal bovine serum (FBS, DMEM, Gibco Biotech, United States) and 0.5% penicillin–streptomycin (PS, DMEM, Gibco Biotech, United States). EDTA (DMEM, Gibco Biotech, United States) treatment was used to detach cells for splitting. During the logarithmic growth phase of BV2 cells, the cell medium was replaced with DMEM without glucose (90,113, Solarbio, Beijing, China). Cells were placed in anoxic chambers containing 5% CO_2_ and 95% N_2_, which were placed in an incubator for 1, 2, 3, 4, 6, or 8 h to establish an OGD model. At the end of OGD, the medium was replaced with high-glucose DMEM for subsequent culture in an aerobic incubator for 12 h to establish the OGD/R model in BV2 cells. Cells in the control group were always cultured under normal conditions. THSWD-contained serum was dissolved in water at various concentrations before co-incubation with OGD/R-induced BV2 cells. Based on literature reports, to explore the effective concentration of THSWD-contained serum that could improve the viability of BV2 cells, OGD/R-induced BV2 cells were co-incubated with 0, 2.5, 5, 10, 15, and 20% THSWD-contained serum, and viability was detected via MTT assay. Therefore, the drug concentration that improves the vitality of BV2 cells is selected. Cells in OGD/R + THSWD group were co-incubated with THSWSD rat serum, and cells in the control (CN) group were co-incubated with the same volume of the corresponding solvent.

### Cell viability assay

The BV2 cells (1 × 104/well) were seeded into 96-well plates for assessment of viability with an MTT kit (Beyotime, Shanghai, China).

### Immunohistochemistry

The fixed rat brain tissue was paraffin-embedded, and sections were made. Then, the following were performed: dewaxing, hydration, antigen repair, closure with normal serum, addition of γ-H2AX antibody (Proteintech, 10856-1-AP, 1:200), staining using DAB, staining again where the color turns blue, gradient dehydration with alcohol, making clear blocked slices, and finally observation (400×, ischemic area or corresponding area) under a microscope.

### Immunofluorescence stainings

Fixed samples were incubated with PBS containing 0.5% Triton X-100 for 20 min. PBS was added and washed three times, followed by blocking with 5% BSA for 30 min. Sections were then washed, and primary antibodies (AIM2, A3356, 1:100, Abclonal, CN; Caspase-1, 22915-1-AP, 1:100, Proteintch, CN; NLRC4, A7382, 1: 100, Abclonal, CN) were added and then incubated overnight at 4°C. A secondary antibody (Cy3 GoatAntiRabbit IgG (H+L) (AS007, 1:200, Abclonal, CN) or FITC Goat AntiRabbit IgG (H+L) (AS011, 1:200, Abclonal, CN)) was added and incubated for 50 min at room temperature (23°C ± 2°C), and cell nuclei were stained with DAPI.

### Enzyme-linked immunosorbent assay

Blood was collected from the rat’s abdominal aorta and left at room temperature for 30 min, and the supernatant was centrifuged to detect IL-1β and IL-18 using an ELISA kit.

### Quantitative real-time PCR

Total RNA was extracted from each group of rat brain tissue according to the instructions of the EZ-10 Total RNA Mini-Preps Kit, reverse transcribed into cDNA, and analyzed using the 2^−∆∆Ct^ method for relative quantification. The primer sequences are shown in Table 3.

**TABLE 3:** Primer sequence.

Primer		Sequence	Product length
Gapdh	Forward	CTC​CAC​TCA​CGG​CAA​ATT​CAA​C	147
	Reverse	GTA​GAC​TCC​ACG​ACA​TAC​TCA​GC	
Polg1	Forward	GAT​GAA​TGG​GCC​TAC​CTT​GA	66
	Reverse	TGG​GGT​CCT​GTT​TCT​ACA​GC	
Tert	Forward	CTA​GCT​CAT​GTG​TCA​AGA​CCC​TCT​T	110
	Reverse	GCC​AGC​ACG​TTT​CTC​TCG​TT	
Dloop1	Forward	CCCTTCCCCATTTGGTCT	45
	Reverse	TGGTTTCACGGAGGATGG	
Dloop2	Forward	CAG​TCA​TAA​ACT​CTT​CTC​T	70
	Reverse	CGG​AGG​ATG​GTA​GAT​TAA​T	
IL-1β	Forward	AGTTGACGGACCCCAAA	104
	Reverse	TCT​TGT​TGA​TGT​GCT​GCT​G	
IL-18	Forward	GGAGGGTTTGTGTTCCAG	62
	Reverse	AAT​ACA​GGC​GAG​GTC​ATC​A	

### Detection of cytosolic deoxyribonucleic acid

After the I/R and ODG/R ended, total DNA and cytosolic DNA were extracted from hippocampal and BV2 cells and assayed based on the literature by Yang et al. (2021). The primers for gDNA are Tert and Plog1, and the primers for mtDNA are Dloop1 and Dloop2.

### Statistical analysis

SPSS and GraphPad software were used to analyze the data. Normal distributed measures were expressed as mean ± standard deviation, and one-way ANOVA and *t*-test were used. *p* < 0.05 was statistically significant.

## Results

### Neurological functional scores

**FIGURE 1:**
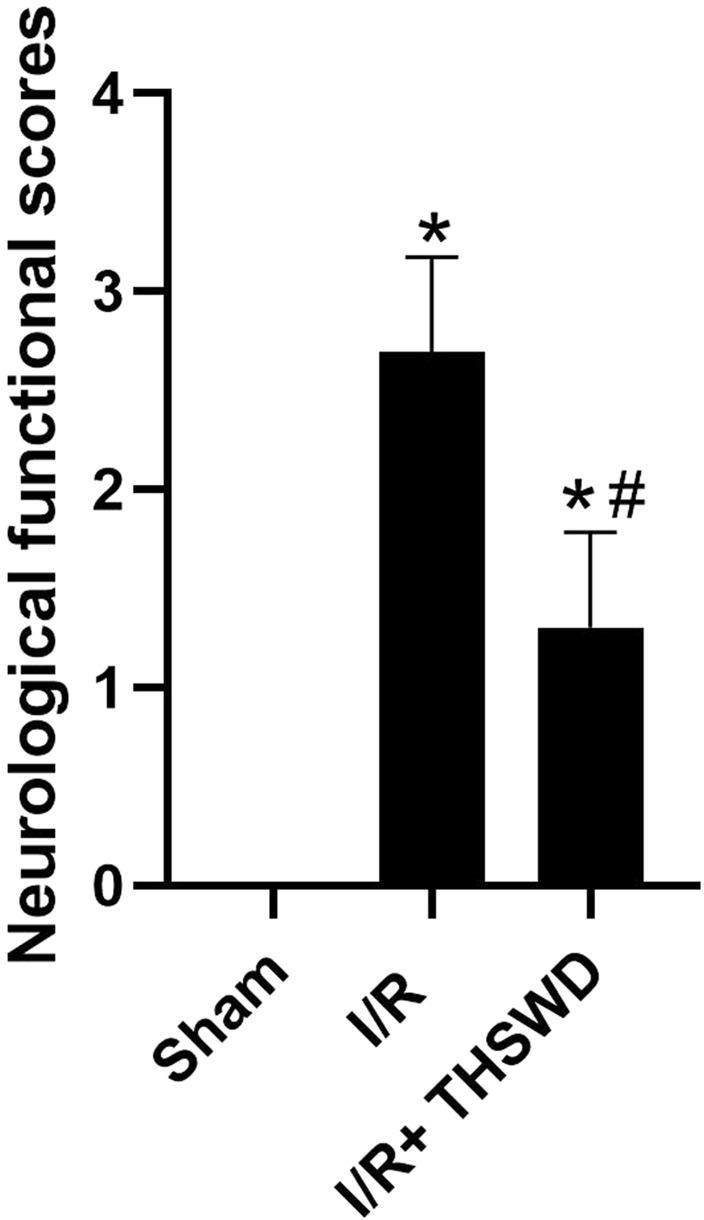
Neurofunctional score analysis of taohong siwu decoction (THSWD). Notes: *p < 0.05 vs. Sham group; ^#^ *p* < 0.05 vs. I/R group.

After the rats were modeled, all rats in all groups except the Sham group had significant neurological deficits, indicating successful modeling, as shown in Figure 1.

### Taohong siwu decoction-contained serum increased the viability of BV2 cells following induction by oxygen-glucose deprivation/reoxygenation

**FIGURE 2:**
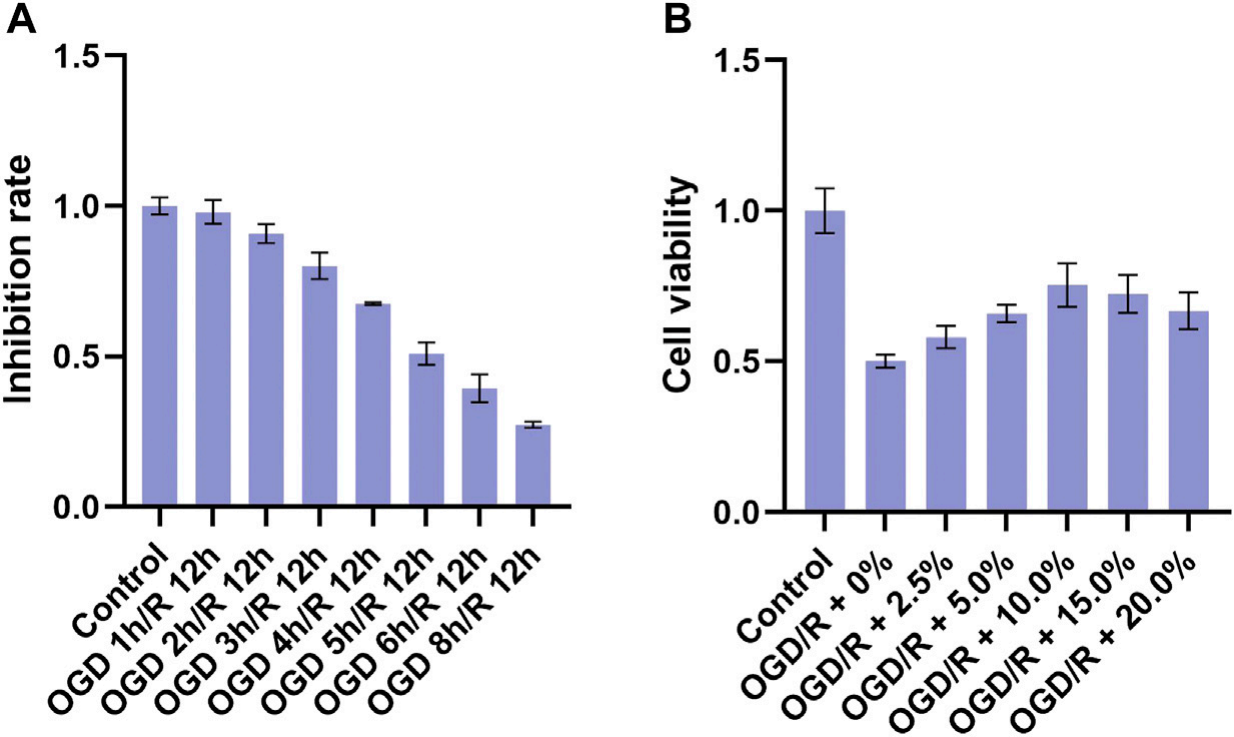
Inhibition rate and different concentrations of THSWD-containing serum. Notes: **(A)** inhibition rate **(B)** cell viability.

To establish a suitable OGD/R model of BV2 cells, BV2 cells were first exposed to OGD for 1, 2, 3, 4, 5, 6, or 8 h and then reoxygenated for 12 h. The MTT assay results (Figure 2A) revealed that OGD 3 h/R 12 h significantly decreased cell viability, whereas OGD 4 h/R 12 h, OGD 5 h/R 12 h OGD 6 h/R 12 h, and OGD 8 h/R 12 h more significantly decreased viability, suggesting excessive cell injury. In addition, OGD 5 h/R 12 h could inhibit nearly 50% cell viability, and we chose it as the best condition. To explore the effective concentration of THSWD-contained serum that could improve the viability of BV2 cells, OGD/R-induced BV2 cells were co-incubated with 0, 2.5, 5, 10, 15, and 20% THSWD-contained serum, and viability was detected via MTT assay (Figure 2B). Among the tested concentrations, cell viability was most obviously improved with 10% THSWD-contained serum. Therefore, we chose 10% THSWD-contained serum as an effective drug concentration for follow-up experiments.

### Taohong siwu decoction reduced the expression of absent in melanoma 2,NLRC4, and Caspase-1 in the ischemic stroke model

**FIGURE 3:**
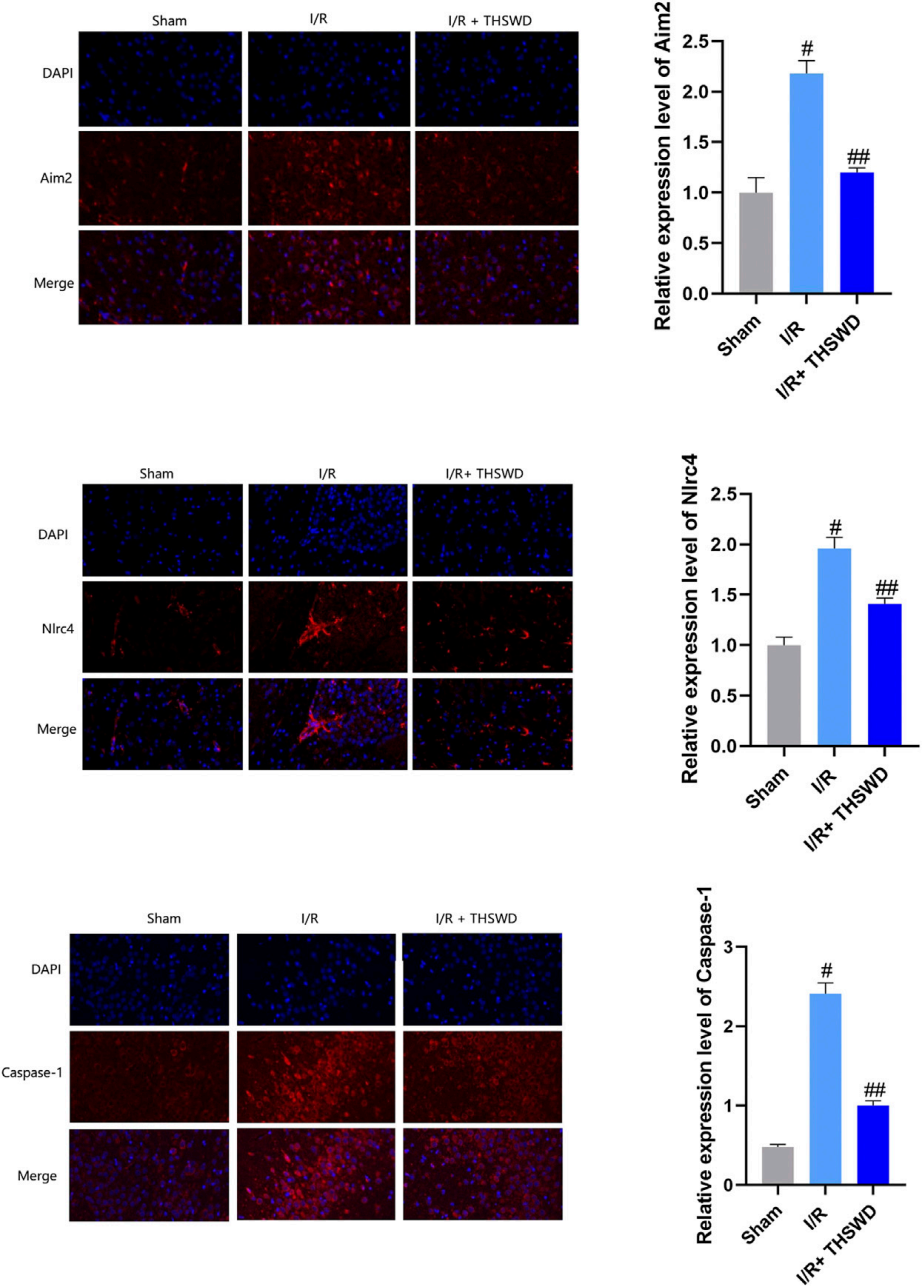
Expression of absent in melanoma 2 (AIM2), NLRC4, and caspase-1 inflammasomes in animal models. Notes: ^#^ *p* < 0.01 vs. Sham group; ^##^ *p* < 0.01 vs. I/R group.

Experimental stroke causes DNA damage, where AIM2 inflammasomes are the main inflammasomes involved in DNA damage, and NLRC4 can form inflammasomes involved in regulating host immune and inflammatory responses (Cui et al., 2000; Guo et al., 2018). AIM2 is activated by the cleavage of procaspase-1, which is cleaved to caspase-1, which is closely associated with the inflammatory form of cell death (Sun and Scott, 2016). The results are shown in Figure 3. By comparison, it was found that the expression levels of AIM2, NLRC4, and Caspase-1 were significantly inhibited in the I/R + THSWD group after the gavage of THSWD to stroke rats.

### Taohong siwu decoction reduced the expression of absent in melanoma 2, NLRC4, and Caspase-1 in oxygen-glucose deprivation/reoxygenation-induced BV2 cells

**FIGURE 4:**
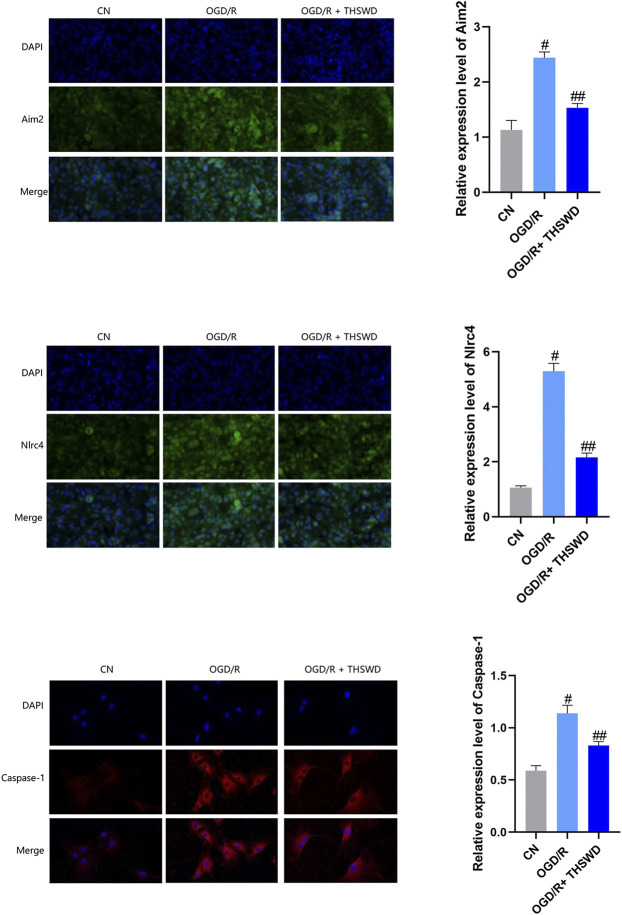
Expression of AIM2, NLRC4 and caspase-1 inflammasomes in cellular models. Notes: ^#^ *p* < 0.01 vs. Sham group; ^##^ *p* < 0.01 vs. I/R group.

To further evaluate the inhibitory effects of THSWD on AIM2 and NLRC4 DNA recognizers and their downstream caspase-1 inflammatory factors, we constructed an OGD/R for BV2 cells and administered THSWD-containing serum for immunofluorescence assays. We found that the expression levels of AIM2, NLRC4, and Caspase-1 were higher in the OGD/R group than in the CN group. After administration of THSWD-containing serum, we found that the OGD/R + THSWD group had a significant inhibitory effect on the high expression levels of AIM2, NLRC4, and Caspase-1, as shown in Figure 4.

### Taohong siwu decoction reduced the release of inflammatory factors from the ischemic stroke model

**FIGURE 5:**
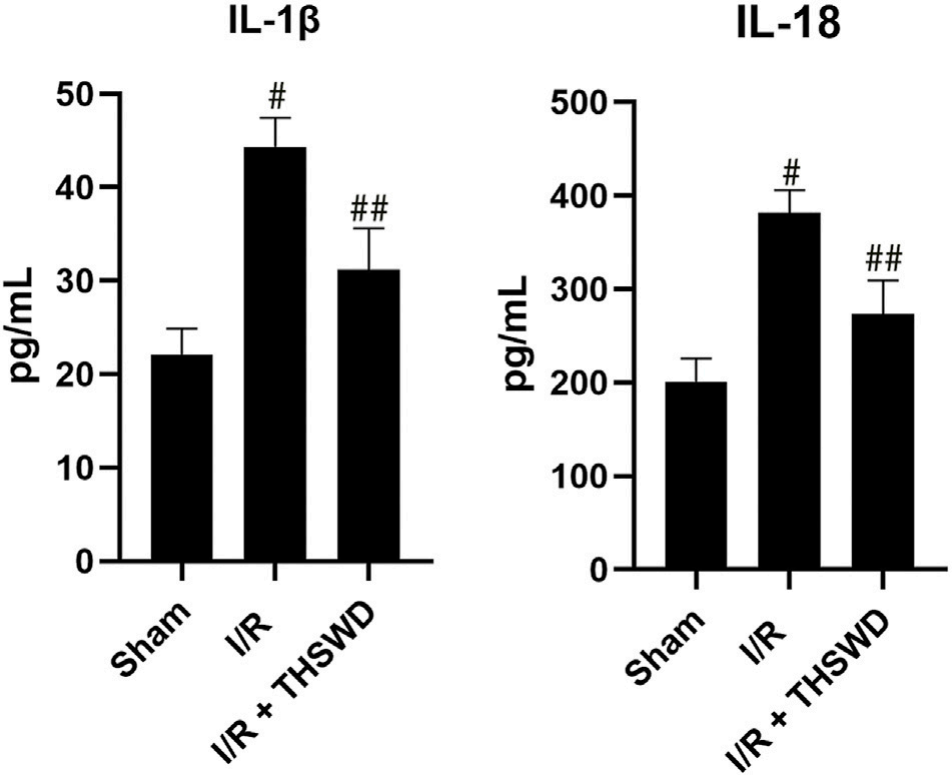
ELISA analysis of THSWD. Notes: ^#^ *p* < 0.01 vs. Sham group; ^##^ *p* < 0.01 vs. I/R group.

Activated caspase-1 is able to activate IL-1β and IL-18 inflammatory factors and induce their release, which in turn recruits other inflammatory cells and amplifies the inflammatory response (Broz and Dixit, 2016). We used ELISA to detect IL-1β and IL-18 inflammatory factors, and the results showed that the levels of IL-1β and IL-18 were significantly increased in the I/R group compared with those in the Sham group and decreased in the I/R + THSWD group compared with those in the I/R group, as shown in Figure 5. THSWD can prevent the release of the above indicators.

### Taohong siwu decoction reduced the release of inflammatory factors from oxygen-glucose deprivation/reoxygenation-induced BV2 cells

**FIGURE 6:**
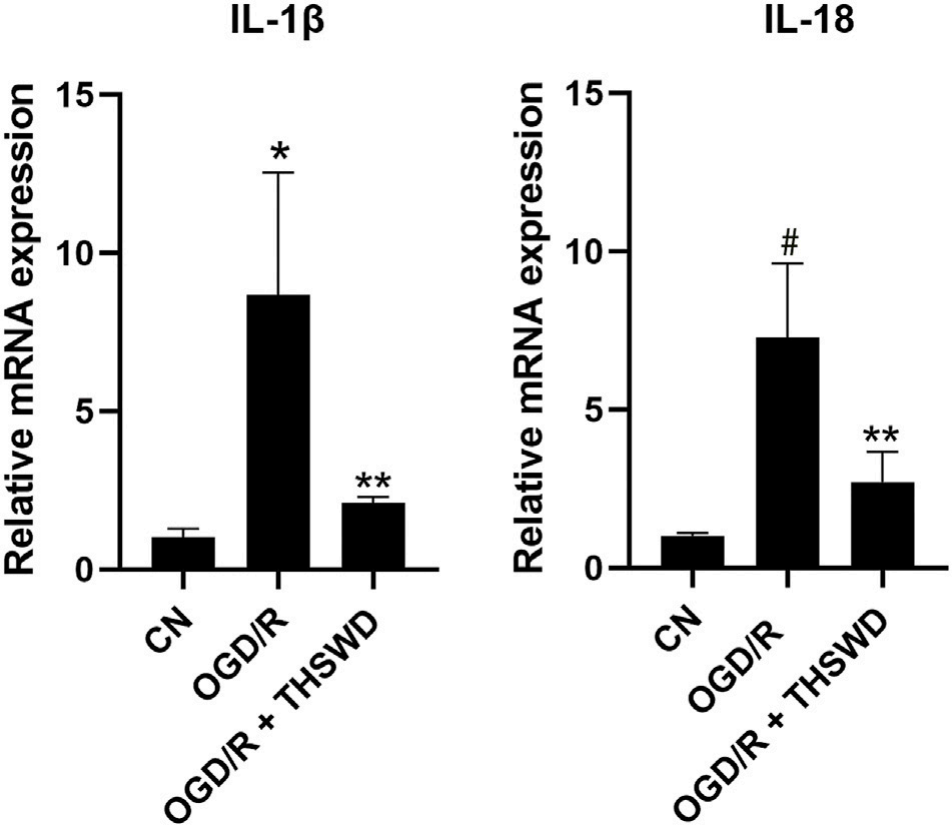
mRNA expression of IL-1β and IL-18. Notes: *p < 0.05, ^#^ *p* < 0.01 vs. Sham group; **p < 0.05 vs. I/R group.

To examine whether THSWD-contained serum could inhibit the inflammatory response of OGD/R-induced BV2 cells, qPCR was performed to detect levels of several major inflammatory factors. The results demonstrated that the release of IL-1β and IL-18 were significantly increased in the OGD/R group compared with those in the CN group but significantly decreased in the OGD/R + THSWD-contained serum group compared with those in the OGD/R group after treatment, as shown in Figure 6. These findings suggest that THSWD-contained serum prevented the inflammatory response of BV2 cells following induction by OGD/R.

### Reduce deoxyribonucleic acid damage

**FIGURE 7:**
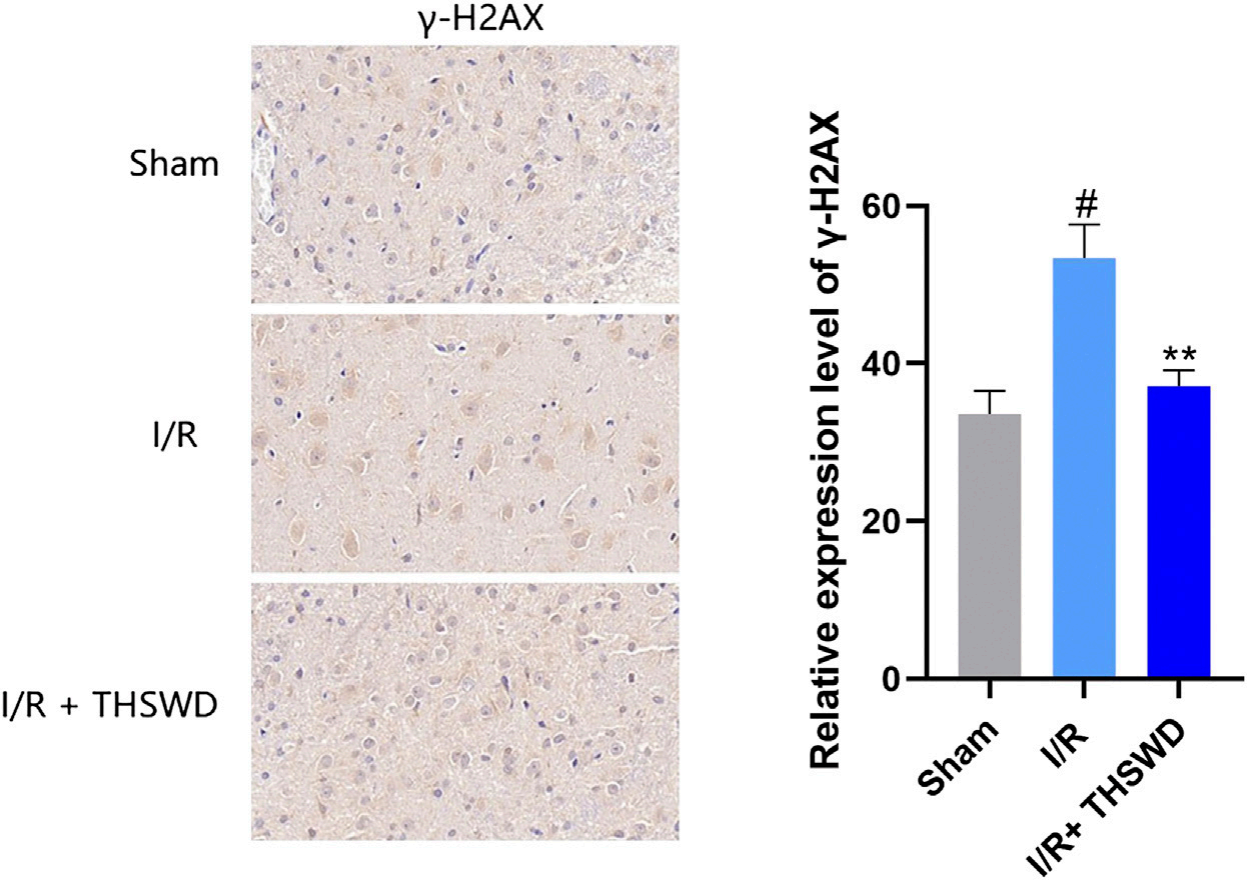
Expression of γ-H2AX in each group. Notes: ^#^ *p* < 0.01 vs. Sham group; **p < 0.05 vs. I/R group.

**FIGURE 8:**
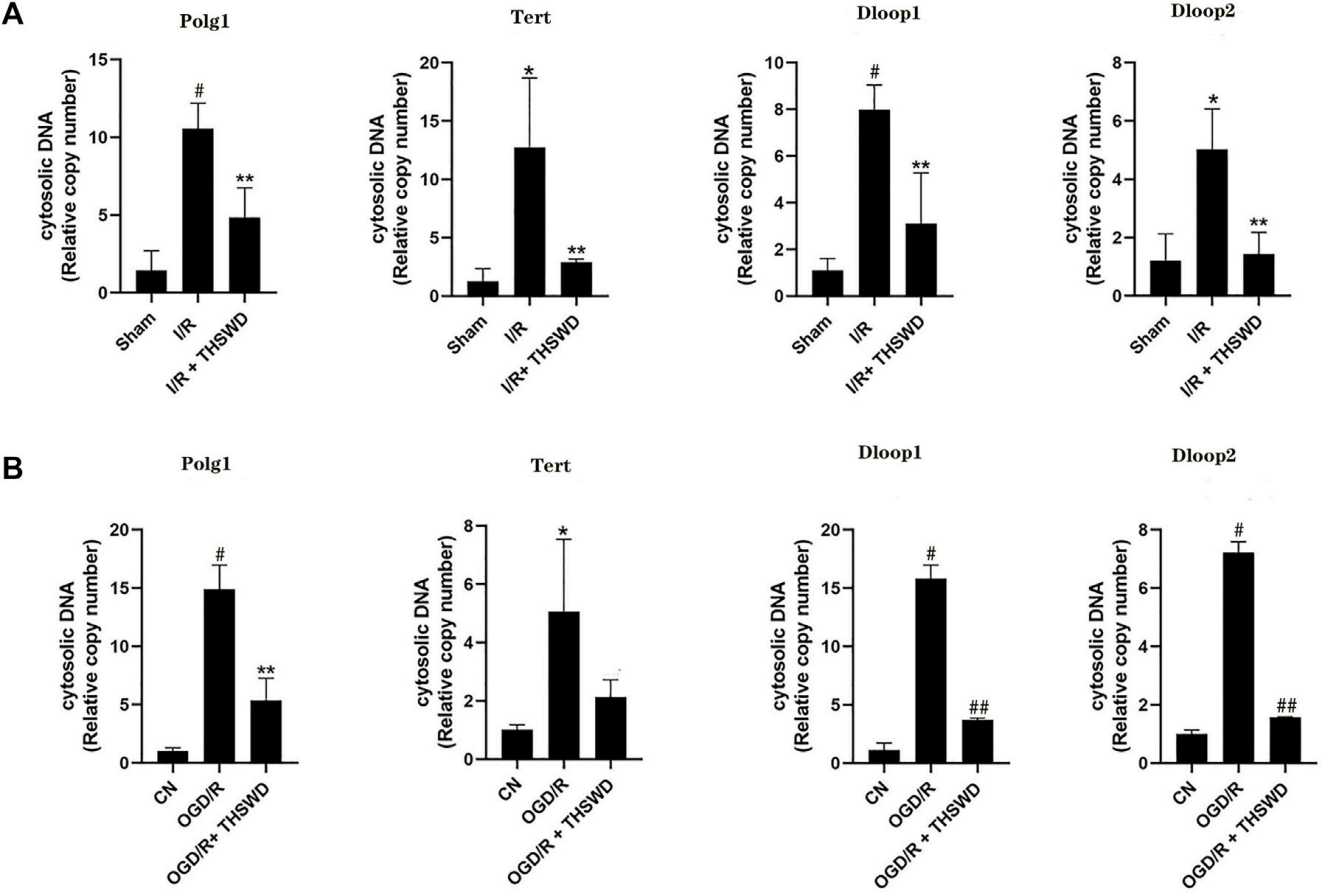
DNA) testing. Notes: **(A)** animal models **(B)** cellular models. A *p < 0.05, ^#^ *p* < 0.01 vs. Sham group; **p < 0.05 vs. I/R group. B *p < 0.05, ^#^ *p* < 0.01 vs. CN group; **p < 0.05, ^##^ *p* < 0.01 vs. oxygen-glucose deprivation/reoxygenation (OGD/R) group.

γ-H2AX is a specific biomarker for characterizing DNA damage, especially DNA double-strand breaks (DSBs) (Lin et al., 2019). Although experimental stroke produces DNA damage, we established a rat stroke model and used immunohistochemistry for the detection of γ-H2AX, a marker of DNA damage. We found that the expression of γ-H2AX was clearly observed to be higher in the I/R group than in the Sham group, and after THSWD treatment, the expression of γ-H2AX was significantly inhibited in the I/R + THSWD group, as shown in Figure 7. Suggesting that THSWD can reduce stroke-induced DNA damage. To further prove that THSWD can reduce DNA damage, DNA was extracted from both animal and cellular models and examined via RT-qPCR, and the results are shown in Figures 8A,B. It can be seen that DNA damage was reduced after the administration of THSWD and THSWD-containing serum.

## Discussion

In the present study, we demonstrated that THSWD attenuated mitochondrial DNA and nuclear DNA damage during stroke; reduced AIM2, NLRC4, and Caspase-1 inflammasomes; and inhibited IL-1β and IL-18 inflammatory factor release after IS. In particular, THSWD may reduce the neuroinflammatory response after cerebral ischemia by ameliorating DNA damage after IS and inhibiting inflammatory somatization.

Injured cells can release free DNA such as mitochondrial DNA and nuclear DNA, which induce an inflammatory response by binding to receptors (Kanou et al., 2021). Inadequate blood flow supply to the brain enhances oxidative stress, resulting in DNA damage (Li et al., 2018). Experimental stroke causes DNA damage, a common precursor event for nerve cell death (Cui et al., 2000). Therefore, DNA damage has become a major focus of stroke research. γ-H2AX is a specific biomarker for DNA damage, especially DNA DSB, and the number of focal points formed by γ-H2AX corresponds to the number of DSB. It has been suggested that IS leads to a significant increase in γ-H2AX injury (Lin et al., 2019). We detected the expression of γ-H2AX in the I/R model and the accumulation of cytoplasmic DNA in I/R and OGD/R cells. It was found that γ-H2AX decreased significantly after THSWD intragastric administration in rats with IS. In the I/R and OGD/R models, there was DNA damage, and damage was reduced after administration of THSWD and THSWD-containing serum.

In most cases, activated NLRs and ALRs recruit a two-component protein called apoptosis-associated spot protein that can induce the protein hydrolysis of pro-IL-1β and pro-IL-18 (Man and Kanneganti, 2015). AIM2 contains two typical domains of the hIN-200 protein family structurally, one of which is the N-terminal thermoprotein domain Pyrindo-main and the other is the C-terminal HIN domain. When abnormal double-stranded DNA is present in the cytoplasm, the HIN domain of AIM2 binds to the cytoplasm double-stranded DNA in a sequence-independent manner. AIM2 can be activated by procaspase-1 cleavage (Ravichandran and Heneka, 2021). Activated caspase-1 cleaves pro-inflammatory cytokines such as pro-IL-1β and pro-IL-18 into mature forms of IL-1β and IL-18 and induces their release, which recruits other inflammatory cells and amplifies the inflammatory response (Broz and Dixit, 2016; Sun and Scott, 2016).

It has been shown that cerebrospinal fluid induces the activation of neurons by AIM2 inflammasomes in patients with traumatic brain injury, suggesting that AIM2 may play a potential pathogenic role in neuronal disease (Adamczak et al., 2014). In addition, the deleterious effect of AIM2 on ischemic brain injury has also been shown in rodent models of stroke (Denes et al., 2015). NLRC4 is both an NLR protein and an inflammasome activator that interacts directly with procaspase-1, which in turn mediates the cleavage of IL-1β and IL-18 precursors, converting them into mature inflammatory factors and ultimately creating a “waterfall effect” that leads to a severe inflammatory response (Duncan and Canna, 2018). Recent studies have further highlighted the role of AIM2 and NLRC4 in post-ischemic pathophysiology (Heinisch et al., 2022). It was found that cerebral ischemia-reperfusion injury induces the activation of AIM2 and NLRC4 inflammasomes, promotes the maturation and release of inflammatory factors, amplifies the inflammatory response, and aggravates brain injury (Li et al., 2020). We established an animal stroke model and gave THSWD treatment. We detected the expression of three inflammasomes, AIM2, NLRC4, and Caspase-1 using immunofluorescence and found that the expression of the three inflammasomes was significantly decreased in stroke rats after THSWD administration. Then, we examined the expression and regulation of AIM2, NLRC4, and Caspase-1 inflammasomes in BV2 cells after OGD/R by THSWD-containing serum and found that THSWD-containing serum significantly inhibited the high expression levels of AIM2, NLRC4, and Caspase-1. Next, we examined the expression of IL-1β and IL-18 inflammatory factors in the I/R model and the OGD/R model using ELISA and RT-qPCR and found a significant increase in the model group and a significant decrease in the administered group, indicating that THSWD can inhibit IL-1β and IL-18 inflammatory factors.

## Conclusion

In summary, our results suggest that THSWD may be effective in the treatment of IS by reducing DNA damage after IS, inhibiting the expression of AIM2, NLRC4, and Caspase-1 inflammasomes and the release of IL-1β and IL-18 inflammatory factors. To provide an experimental and theoretical basis for further study of THSWD treatment of IS.

## Data Availability

The original contributions presented in the study are included in the article/supplementary material; further inquiries can be directed to the corresponding authors.

## Abbreviations

THSWD, taohong siwu decoction; IS, ischemic strokes; I/R, ischemic stroke model; OGD/R, oxygen-glucose deprivation/reoxygenation; DNA, deoxyribonucleic acid; AIM2, Absent in melanoma 2; DSB, double-strand break
